# Common contextual influences in ambiguous and rivalrous figures

**DOI:** 10.1371/journal.pone.0176842

**Published:** 2017-05-01

**Authors:** Marouane Ouhnana, Ben J. Jennings, Frederick A. A. Kingdom

**Affiliations:** McGill Vision Research Unit, Department of Ophthalmology, Montreal General Hospital, Montreal, Quebec, Canada; State University of New York Downstate Medical Center, UNITED STATES

## Abstract

Images that resist binocular fusion undergo alternating periods of dominance and suppression, similarly to ambiguous figures whose percepts alternate between two interpretations. It has been well documented that the perceptual interpretations of both rivalrous and ambiguous figures are influenced by their spatio-temporal context. Here we consider whether an identical spatial context similarly influences the interpretation of a similar rivalrous and ambiguous figure. We developed a binocularly rivalrous stimulus whose perceptual experience mirrors that of a Necker cube. We employed a paradigm similar to that of Ouhnana and Kingdom (2016) to correlate the magnitude of influence of context between the rivalrous and ambiguous target. Our results showed that the magnitude of contextual influence is significantly correlated within observers between both binocularly rivalrous and ambiguous target figures. This points to a similar contextual-influence mechanism operating on a common mechanism underlying the perceptual instability in both ambiguous and rivalrous figures.

## Introduction

Our perception of the visual world, while in most situations stable and close to veridical, is sometimes ambiguous and unstable. The Necker cube is a well-known example of an ambiguous figure—a skeleton cube whose perceptual interpretation alternates between facing upwards or downwards [1]. Another form of ambiguity is binocular rivalry, in which different stimuli presented to the two eyes alternate perceptually in dominance [2]. Usually it is the percept of the two stimuli that alternates, but in some cases one perceives a piecemeal mosaic of each eye’s input [3].

The perceptual instability experienced in both ambiguous and rivalrous stimuli has been argued to arise through competition and inhibition between the neurons sensitive to each stimulus, via feedforward, feedback, or as recently suggested, a hybrid of these mechanisms see [4] for a review on ambiguous figures, and [2,5,6] for reviews on binocularly rivalrous figures.

Various manipulations alter the perceived interpretation of both ambiguous and rivalrous figures. These manipulations range from the effects of adaptation to temporal priming [7,8] and extend to manipulations such as learned associations [9]. Long et al. [7] showed that when an *unambiguous* version of an ambiguous figure is presented prior to an ambiguous target, the perceptual interpretation depends on the length of time the former figure is presented. When briefly presented, a priming effect causes the interpretation of the ambiguous target to shift *towards* that of the previously inspected unambiguous figure. However when the prior unambiguous figure is presented for an extended length of time, the interpretation of the figure is shifted *away* from that of the adapted figure. Haijiang et al. [9] demonstrated that a particular interpretation of an ambiguous Necker cube figure could be cued by the motion or position of a disambiguated Necker cube, or even by a sound, that had been previously associated with that interpretation.

Not surprisingly, simultaneous contextual influences also alter the perceptual interpretation of both ambiguous and rivalrous figures [3,10–12], and are thus considered to be valuable tools for probing their underlying mechanisms [13]. Intaitė et al. [10], using a display of overlapping skeleton squares which alternated between being perceived as stacked upwards or stacked downwards, showed that when the squares were flanked by unambiguous coherently stacked versions, observers’ first percepts were biased towards that of the flanking context. The influence of spatial context on ambiguous figures has also been revealed through the introduction of backgrounds favoring a particular percept [14], or when the context itself is ambiguous [15]. Using a Necker cube as stimulus, Dobbins and Grossmann [15] reported that the variability of first percept reports, which favored the view-from-above bias, was reduced when the target Necker cube was presented in an array of other similarly ambiguous cubes than when presented in isolation.

For binocularly rivalrous figures the influence of context has been studied using a variety of paradigms. Kovacs et al. [3] reported that when individual grids of red and green dots were presented dichoptically, the resulting percept alternated between the two colours. When half of the coloured dots were swapped across the two grids, the resulting percept was a grid defined by a single color. Sobel and Blake [12] studied the influence of global coherence using a dichoptic display of four gratings, where one of these rivaled with a rotating radial checkerboard. The non-rivalrous flanker gratings drifted in motion directions that were either consistent or inconsistent with a coherent global percept. When the context motion was consistent with a global percept, the predominance of the grating percept in the rivalling pair was found to be significantly higher. The effect of global coherence in binoculary rivalrous displays has also been shown to occur using combinations of monocularly rivalrous figures. Vergeer et al. [16] presented a shape-from-motion-defined cylinder with the leftward drifting dots to one eye and the rightward drifting dots to the other. Observers predominantly reported perceiving a cylinder rather than a convex or concave shell of horizontally drifting dots.

In this communication, we ask whether such simultaneous contextual influences operate similarly for both ambiguous and binocularly rivalrous stimuli, implying a common context-influence mechanism. Our study serves also to address the wider issue of whether the two forms of perceptual instability are mediated by a common underlying mechanism.

While it is tempting to draw parallels between the contextual influences observed in both rivalrous and ambiguous figures, it is important to note that the contexts described in the literature for both classes of figures are never the same. In the case of ambiguous figures, the contexts are always unambiguous in interpretation, whereas with binocularly rivalrous figures the contexts are invariably one component of the competing stimuli. Thus in order to investigate whether context has a similar influence on ambiguous and rivalrous figures one has to equate both the context figures as well as the perceptually unstable figures themselves.

When a rotating Necker cube is observed, the perceptual experience is of a skeleton cube rotating in a clockwise or counter-clockwise motion direction. A binocularly rivalrous version of this figure can be created by presenting clockwise and counter-clockwise rotating unambiguous skeleton cubes to each eye. The perceptual experience of this rivalrous skeleton cube is similar to that of its ambiguous Necker cube counterpart, that is, its rotation appears to fluctuate from clockwise to counter-clockwise and back. Importantly, although the visual experience of our rivalrous skeleton and ambiguous Necker cube are comparable, the physical properties of the figures differ in crucial ways: one is defined by a dichoptic pair of non-ambiguous figures, while the other is defined by a single binocular ambiguous figure.

In the current study we investigated whether the introduction of an identical spatial context similarly influenced the perceptual interpretation of rivalrous and ambiguous stimuli when both context, rivalrous and ambiguous figures are equated. A paradigm similar to Ouhnana and Kingdom [11] was employed to compare the degree of coherence between the target and context figures. In their study, observers were instructed to report the motion direction of a rotating Necker cube in the context of an unambiguous rotating skeleton cube that changed motion direction randomly throughout the trials. Through a procedure akin to reverse correlation, a Phi coefficient was computed that described the extent to which the context influenced the perceived motion direction of the target. A similar procedure is used here.

## General methods

### Subjects

Five observers participated in both experiments, two where authors (MO and BJ), while the others were naïve to the experimental aims. All had normal or corrected-to-normal visual acuity. Written consent was obtained for all participants and all experimental protocols were approved by McGill University Research Ethics Board.

### Apparatus

The stimuli were created using Blender (version 2.67b), an open-source 3D computer graphics rendering environment, and displayed using Psychophysics Toolbox (Version 3.12) running under Matlab (MathWorks Inc., version 8.1). The stimuli were presented on a ViewSonic G225f Graphic Series CRT monitor connected to an Apple Mac Pro. The display was driven at 60 Hz with a resolution of 1024x768.

Participants viewed the display through a modified Wheatstone stereoscope, which employed four front-surfaced mirrors per eye, with an effective viewing distance along the light path of 55 cm. The stereoscope had a visible window (to each eye) of approximately 9.8 deg x 12.4 deg.

### Stimuli—General

The stimuli consisted of rotating unambiguous skeleton cubes and rotating ambiguous Necker cubes. The difference between the skeleton and Necker cubes was that in the skeleton cubes the back parts of the skeleton were occluded by the front parts, rendering the cubes unambiguous. The skeleton cubes were used for the contexts and for the binocularly rivalrous targets, while the Necker cubes were used as the ambiguous targets. Depending on the experimental condition, the context figure was either presented as a single or a pair of figures, where the pair of figures were identical. The rivalrous skeleton cubes were always presented in pairs regardless of the experimental condition. Each of the rotating skeleton cubes was presented with an opposite rotation direction. In other words, if the cube was facing upwards in one eye and rotating clockwise, the other eye would be presented with a cube facing downwards with an anti-clockwise motion direction. The ambiguous pair was either presented as an identical pair, one to each eye, or as single figure presented to one eye. All cubes measured ~3 x 3 degrees of visual angle. All figures rotated at a quarter of a revolution per second and all animations were rendered at 60 frames per second. Orthogonal projection was used for all stimulus conditions, i.e. there were no perspective cues.

#### Stimuli—Ambiguous Necker cube

**Fig 1:**
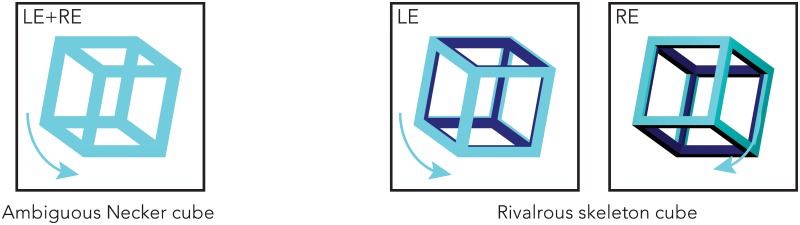
Example of the ambiguous Necker cube (left) and binocularly rivalrous skeleton cube (right pair). One of the skeleton cubes also served as the context figure. Orthographic projection was used to render ambiguous the motion direction of the Necker cube, while the addition of shading and color difference disambiguated the motion directions of skeleton cubes.

The ambiguous Necker cube, shown on the left of Fig 1, was created using Blender meshes with materials set to teal with shading turned off to maintain ambiguity. The Necker cube was presented either as a single figure or a pair of figures depending on the experimental condition. The figure’s XYZ rotational space was set to [20,0,0], with Z dictating the cube’s rotation. Blender XYZ rotational space corresponded to roll, pitch, and yaw, where yaw dictated the figure’s rotation around its vertical axis, each trial starting at zero.

#### Stimuli—Context and binocularly rivalrous skeleton cubes

For the context and rivalrous skeleton cubes, as shown on the right of Fig 1, the figures were created using Blender meshes with materials set to teal for their outer surfaces and dark blue on their inner surfaces. Due to the orthographic projection used to render the stimuli, the external and internal surface color differences added occlusion information serving to disambiguate the figure’s motion direction (clockwise or anti-clockwise). The figure’s XYZ rotational space was set to [20,0,0], with Z dictating the cube’s rotation.

### Experimental conditions & procedures

**Fig 2:**
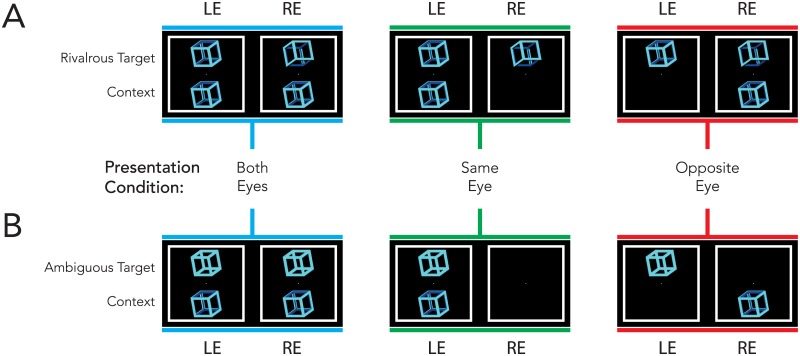
Sample frames illustrating each context and target condition for the rivalrous target (top), and ambiguous target (bottom). The context figure was presented either to both eyes (left hand side), the same eye (middle), or the opposite eye (right hand side) of the target. For each condition, the context was presented either above or below the fixation dot.

There were three eye presentation conditions for both types of target (ambiguous Necker cube and rivalrous skeleton cube), as illustrated in Fig 2. In addition the context was positioned either above or below fixation. The three eye presentation conditions were as follows: 1. A pair of identical context figures presented binocularly—see the leftmost column of Fig 2 highlighted in blue. 2. A single context figure presented monocularly: in the case of the rivalrous skeleton cube this was to the same eye as the test skeleton cube that matched the context’s motion direction—see the top middle column of Fig 2 highlighted in green; in the case of the ambiguous Necker cube this was to the same eye as the monocularly presented ambiguous target—see bottom middle column of Fig 2 highlighted in green. 3. A single context figure presented monocularly: in the case of the rivalrous target this time to the opposite eye as the skeleton cube that matched the context’s motion direction—see the top right of Fig 2 highlighted in red; in the case of the ambiguous Necker cube this time to the opposite eye as the monocularly presented ambiguous target—see the bottom of the rightmost column of Fig 2 highlighted in red.

Observers were instructed to report via a key-press when the context and target were perceived to rotate in the same motion direction and by a different key press when they were perceived to rotate in opposite motion directions. The motion direction of the context figure was randomly selected at the start of every trial. During each of the 30 s trials, there was a 0.5 probability that a change in context motion direction would occur when the context was aligned fronto-parallel. The context and target figure were either swapped across eyes (experiment 1) or remained in the same eye (experiment 2). All conditions were interleaved and 10 repetitions were performed.

## Results

### Analysis

For both Experiments 1 and 2, an analysis of the magnitude of contextual influence was first analyzed in terms of the concordance between the motion direction of the context figure and the perceived motion direction of the target across each trial. This was accomplished through a method analogous to reverse correlation, as in our previous study (Ouhnana & Kingdom, 2016). The magnitude of influence was calculated using the Phi coefficient, which is a measure of association between two binary variables. The two variables in our study were the *clockwise* and *counter-clockwise* motion, or perceived motion direction of respectively the context and target figure.

**Fig 3:**
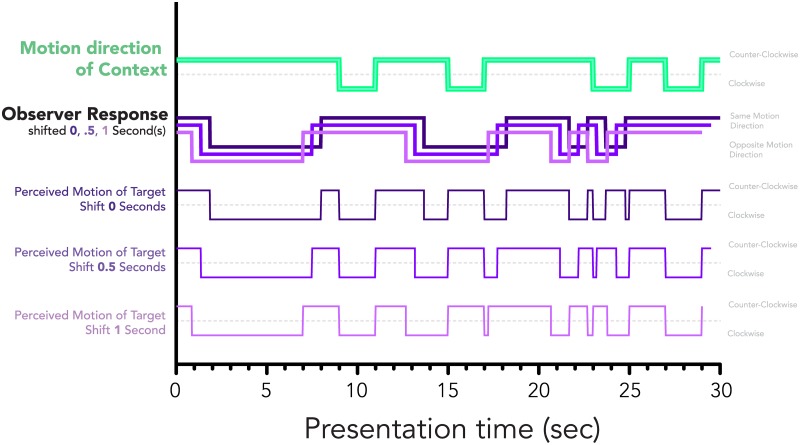
Example of context motion sequence (top), observer response (second), and extracted perceived motion of the target sequence (three from the bottom) taken from a sample trial for three example shifts of the timeline.

To extract the perceived target motion direction from the observer responses, the reported motion directions (same versus opposite) throughout each trial were compared to the clockwise versus counter-clockwise motion direction timeline of the context figure. An example context motion sequence (top), reported observer response (second from the top), and extracted perceived target motion sequence (three from the bottom) is illustrated in Fig 3.

Prior to the Phi coefficient analysis, the observer response timeline was shifted by various amounts to account for any observer response delays, prior to extracting the perceived motion direction timeline of the target. The shifts of the response timeline were in multiples of approximately 15 milliseconds, or a single frame presentation, up to a maximum of 1 second, or 60 presentation frames. Since Phi coefficient analyses require balanced data sets, we truncated the context timeline by the amount of shift applied to the response timeline. Examples of shifts applied to observer responses and resulting extracted perceived target motion directions are illustrated in Fig 3. In the figure, shifts of 0s, 0.5s, and 1s are shown both in terms of the change to the observer response sequence and to the resulting extracted perceived motion direction of the target figure for each of the given shifts.

Following these shifts, the motion direction of the context was compared to the extracted perceived motion direction of the target, and the number of instances of the four possible pairings of responses and context entered into Table 1. From the possible Phi coefficients, i.e. for each of the possible shifts, the maximum coefficient was taken and retained.

**Table 1:** 

	Target perceived clockwise	Target perceived counter-clockwise	total
**Context clockwise**	n_11_	n_10_	n_1·_
**Context counter-clockwise**	n_01_	n_00_	n_0·_
**total**	n_·1_	n_·0_	n

The Phi coefficient *ϕ* was calculated as follows:

$$\phi =\frac{n_{11}n_{00}-n_{10}n_{01}}{\sqrt{n_{1\cdot }n_{0\cdot }n_{\cdot 0}n_{\cdot 0}}}$$ (1)

From the equation one can see that a Phi coefficient of 1 indicates a perfect correspondence between the perceived target and the context motion direction. The Phi coefficient values were then averaged across conditions and then correlated between the ambiguous and rivalrous figures.

### Experiment 1: With interocular swaps

When a rivalrous pair is swapped across eyes, the previously suppressed percept escapes inhibition resulting in a single perceptual change [17]. On the other hand when a monocular ambiguous figure is swapped across eyes there is a significant increase in the subsequent *rate* of perceived reversals in comparison to normal binocular presentation (Spitz & Lipman, 1962). The changes in interpretation observed in the rivalrous figure following an eye swap are thus not equivalent to the increase in reversal rates observed in an ambiguous figure: the former is a reversal in interpretation that is temporally tied to the swap of the rivalrous pair, while the latter is a general increase in reversal rates throughout inspection of the ambiguous figure. We were therefore curious as to the effect of this manipulation with our stimulus protocol. Following a motion direction change of the context figure, both context and target were swapped between eyes, unaware to our observers.

**Fig 4:**
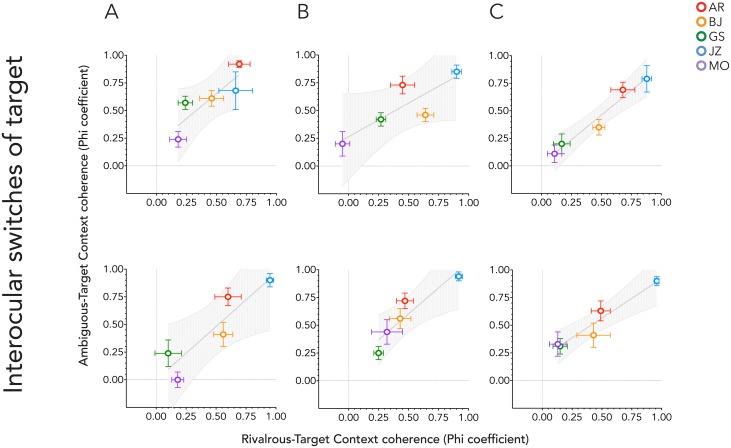
Mean Phi coefficients for context-target coherence averaged across trials. Each graph plots the bi-stable target coefficients against the rivalrous target coefficients. Different graphs are for different context condition. A, context presented to both eyes; B, context presented to the same eye as the matched target; C, context presented to the opposite eye as the matched target. The upper panel is for when the target figure was presented above the fixation cross, the lower panel when below it.

The mean context-target Phi coefficient for each observer and target condition was calculated to assess the magnitude of contextual influence. The Phi coefficients for the ambiguous Necker and rivalrous skeleton cubes were then correlated using Pearson’s correlation coefficient *r*, and are summarized in Table 2 and plotted in Fig 4, along with *p* values that determine whether the values of *r* are significantly different from zero. Contextual influences were found to be highly and significantly correlated between target types for all conditions.

**Table 2:** Correlations r between the mean Phi coefficients of the ambiguous and rivalrous targets for Experiment 1.

Condition	Target Above		Target Below	
	*r*	*p*	*r*	*p*
Both eyes	0.86	<.05	0.91	<.05
Same eye	0.84	<.05	0.93	<.05
Opposite Eye	0.98	<.01	0.96	<.01

These significant correlations are found regardless of the temporal shifts introduced to take into account any observer response delays. The mean ratio of shifted to non-shifted Phi coefficients across both the ambiguous and rivalrous conditions was 1±0.02 (mean±SEM); in other words the shifts had a minimal effect on the coefficients. Furthermore, the shifts had even less impact on the correlation of the magnitude of context influence between the rivalrous and ambiguous target: the mean ratio change was 1±0.005 (mean±SEM).

### Experiment 2: Effect of eye dominance

With rivalrous figures, the interpretation in the dominant eye tends to dominate [18]. Since the first experiment involved swapping the context and target across the eyes following a context motion direction change, it would seem prudent to investigate the effect of eye-dominance on the contextual influences studied here. We employed a similar methodology to that of Experiment 1 with the added differences that: 1. the location of the context and target was *not* swapped across eyes following a change in context motion direction; 2. the ambiguous target or the rivalrous skeleton cube that matched the context’s motion direction was presented in separate conditions to the observer’s dominant and non-dominant eye.

Eye dominance for each observer was measured using the Miles test [19]. For the ambiguous Necker cube condition in which the context figure is presented to both eyes, the manipulation of eye-dominance is not possible. In this case the data is that from Experiment 1.

**Fig 5:**
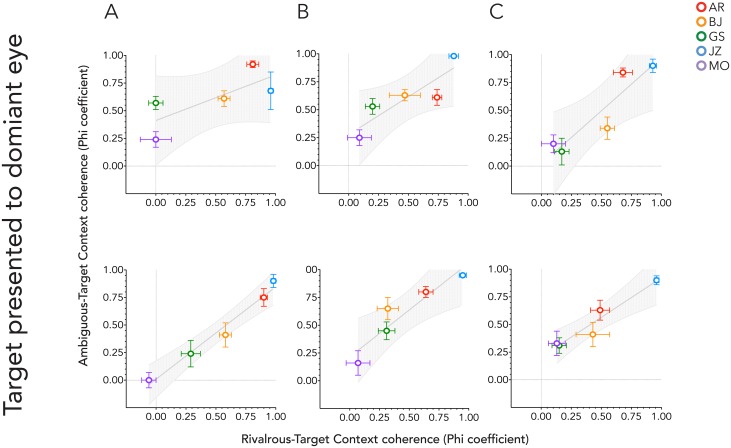
Mean Phi coefficients for the bi-stable target condition are plotted against those obtained for the rivalrous target condition by each context condition. The matched target was always presented to the observer’s dominant eye. A, context presented to both eyes; B, context presented to the same eye as the matched target; C, context presented to the opposite eye as the matched target. The upper panel is for when the target was presented above the fixation cross, the lower panel when below it.

**Fig 6:**
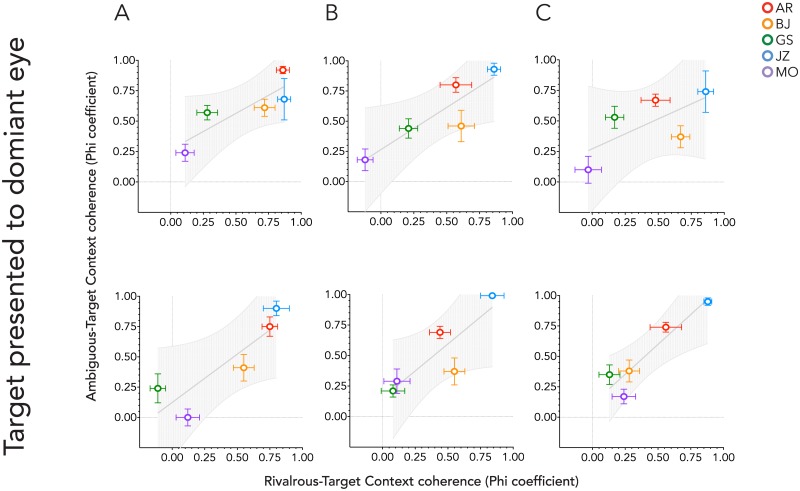
Mean Phi coefficients for the ambiguous target are plotted against those for the rivalrous target, for each context condition. The matched target was always presented to the observer’s non-dominant eye. A, context presented to both eyes; B, context presented to the same eye as the matched target; C, context presented to the opposite eye as the matched target. The upper panel is for when the target figure was presented above the fixation cross, the lower panel when below it.

The correlations between the Phi coefficients for the ambiguous and rivalrous data are summarized in Table 3 and plotted in Figs 5 and 6 for the target to dominant eye and target to non-dominant eye, respectively. Contextual influences were found to be highly correlated between target types for all except for when the target was presented to both eyes above fixation in the dominant eye condition where the correlation was highly correlated and approached significance, *p* = 0.07. As with Experiment 1 the addition of the delay had a negligible effect on the Phi coefficient values (ratio change 1.09 ±0.11 (mean±SEM)) or subsequent correlations between ambiguous and rivalrous figures (mean ratio change 1 ±0.003 (mean±SEM).

**Table 3:** Correlations r between the mean Phi coefficients of the ambiguous and rivalrous targets for Experiment 2.

Condition	Target Above		Target Below	
	*r*	*p*	*r*	*p*
	Target to dominant eye			
Both eyes	0.76	0.07	0.99	<.01
Same eye	0.88	<.05	0.94	<.01
Opposite Eye	0.92	<.05	0.88	<.05
	Target to non-dominant eye			
Both eyes	0.86	<.05	0.91	<.05
Same eye	0.84	<0.01	0.93	<.05
Opposite Eye	0.98	<0.01	0.96	<.01

## Discussion

The results with the Necker cube confirm our previous report (Ouhnana & Kingdom, 2016) and extend it to a binocularly rivalrous figure. Our goal in this study however was to determine whether spatial contextual information similarly influenced the perceptual judgements of both types of stimuli, when designed to elicit a similar perceptual experience. The data show that the context influenced both classes of stimuli similarly: the magnitude of contextual influence between rivalrous and ambiguous target conditions was positively and significantly correlated across observers for nearly all experimental conditions tested. It has been argued that similarities between different types of perceptually unstable figure, such as binocular and monocular rivalry, reflect similar or even identical disambiguation mechanisms [20]. The findings of the present study strongly support this idea and extend it to include ambiguous figures such as the Necker cube.

This might at first seem surprising. The perceptual experience of rivalrous and ambiguous figures differs markedly even when subjected to similar manipulations. For example, when a rivalrous figure is swapped across eyes, the suppressed interpretation assumes dominance [17]. Swapping a Necker cube from one eye to the other on the other hand, which if it produced a switch in interpretation would result in reversal rates being more-or-less constant across time, instead results in an increase over time in reversal rates when compared to binocular viewing [21].

However, these differences between rivalrous and ambiguous figures are arguably bottom-up influences, whereas the present study focuses on what is presumably the top-down influence of context. We argue that the high correlations found in this study are due to the overriding influence of the context figures. There are good examples in the literature where contextual influences overcome competing manipulations such as in the study described by [10]. They demonstrated that the influence of spatial context, which biases the interpretation of an ambiguous figure *towards* that of context, overcame any adaptation effects which on their own bias the interpretation of the target figure *away* from that of the adaptor figure.

Although the competing interpretations in perceptually unstable figures, whether produced by rivalry or ambiguity, are known to be influenced by contextual information, the contexts employed in previous studies for the two types of figure have been very different. In previous rivalry studies, the context perceptually combined with the rivalrous target to form a globally coherent percept, biasing the interpretation towards that of the global percept. A good example of this is the study by Sobel and Blake [12] described in the Introduction. On the other hand in previous ambiguous figure studies, the spatial context was an independent figure, albeit one that also biased the target’s percept towards that of the context [10]; [11]). In the present study, we demonstrated that an independent context, i.e. one that does not form a globally coherent percept with the target, influences the perceptual interpretation of a rivalrous figure similarly to that of its ambiguous counterpart.

Ouhnana & Kingdom [11] argued that the Gestalt grouping principles of common-fate and, importantly, change synchrony underpinned the perceptual binding they observed between a simultaneously presented rotating context and Necker cube. The principle of common fate is that objects that *move* together are perceptually bound together, while the principle of change synchrony is that objects that *change* together are perceptually bound together [5,22]. In the current study, we found that these binding principles also extend to a rivalrous stimulus that perceptually resembled a Necker cube, and furthermore that the magnitude of perceptual binding was significantly correlated between the two types of figure.

One might think that the perceptual instability in the rivalrous figure was not due to competing monocular interpretations but instead due to ambiguity in a single, binocularly-fused cube, and that this was the reason for the similarities of contextual influence. To test this possibility, we created a demo in which each of the dichoptically presented cubes were defined by a different colour. The changes in perceived motion direction were always observed to be accompanied by changes in color. If the alternations were due to ambiguity in a single fused cube, then one would expect that the reversals would not be tied to the underlying cube colors. We therefore conclude that the changes in interpretation experienced in our rivalrous target are due to binocular rivalry between competing monocular percepts—not ambiguity within a single fused percept.

In our first experiment, both context and target figures were swapped between eyes following a motion direction change of the context figure. This manipulation was undertaken to maximize differences between the two types of targets, in order to assess any potential differential influence of the context. However, because we found no difference between the results of this experiment and the subsequent one in which we did not introduce an eye-swap, we conclude that this manipulation had no effect.

Our second experiment included an examination of the effects of eye dominance. In previous studies, when the rivalrous figures were presented to the same locations in both eyes, the perceptual interpretation was biased towards the input in the dominant eye [18]. In our study we found significant correlations between the different types of figure regardless of the eye-dominance relationships between target and context.

In conclusion, our results argue that both our rivalrous and ambiguous rotating targets share a common contextual-influence mechanism. This finding has a corollary in that it also argues for a common underlying mechanism mediating the perceptual instability of ambiguous and rivalrous figures.
